# Weight adjusted waist index is a superior obesity index for predicting arterial stiffness in type 2 diabetes mellitus

**DOI:** 10.1038/s41598-025-17715-6

**Published:** 2025-08-29

**Authors:** Shijun Gong, Jing Mao, Quan Zhou, HaiFeng Zhou, Qin Liu, Ting Sun, Shenglian Gan

**Affiliations:** 1 Department of Ultrasound, Changde Hospital, Xiangya Medical College of Central South University, Changde, China; 2 Department of Science and Education, Changde Hospital, Xiangya Medical College of Central South University, Changde, China; 3 Department of Endocrinology, Changde Hospital, Xiangya Medical College of Central South University, Changde, China

**Keywords:** Obesity, Weight-adjusted-waist index, Visceral fat, Arterial stiffness, Brachial-ankle pulse wave velocity, Type 2 diabetes mellitus, Endocrinology, Endocrine system and metabolic diseases, Diabetes, Type 2 diabetes

## Abstract

Obesity is a key risk factor for arterial stiffness (AS) and a strong predictor of cardiovascular diseases (CVD). In patients with type 2 diabetes mellitus (T2DM), the risk of AS is further exacerbated due to metabolic dysregulation and chronic inflammation, especially when both conditions coexist. Traditional obesity indices such as Body Mass Index (BMI) and Waist Circumference (WC) have limitations in assessing fat distribution and metabolic risk. New indices like Weight-adjusted Waist Index (WWI), A Body Shape Index (ABSI), and Body Roundness Index (BRI) have been proposed as supplementary or alternative measures. However, the association between WWI and AS in patients with T2DM has not been sufficiently studied. This study aims to evaluate the association between WWI and AS in T2DM patients and compare the diagnostic performance of WWI with traditional obesity indices (BMI, WC) and other new indices (ABSI, BRI) in predicting AS. 1406 patients with type 2 diabetes mellitus (T2DM) were included in this cross-sectional study. Obesity indices were calculated using anthropometric parameters. Brachial-ankle pulse wave velocity (BaPWV) was measured to determine AS. Multivariate logistic regression analysis was used to assess the correlation between the five obesity indices and AS. Smoothed curve fitting was used to test the linear correlations. The diagnostic accuracy of each obesity index was evaluated using receiver operating characteristic (ROC) analyses. Additional subgroup analyses and tests for interaction were conducted. The average BaPWV was 1611.98 (318.81) cm/s. Multivariate logistic analysis indicated a strong correlation between WWI and AS, with each standard deviation increase in WWI resulting in a 48.59 cm/s rise in BaPWV (95% CI 31.66–65.52) and a 44% higher risk of AS (OR = 1.44, 95% CI 1.21–1.70). ROC analysis indicated that WWI identified AS more accurately than the other obesity indices, with an area under the curve (AUC) of 0.659. The AUC for WC, BMI, ABSI, and BRI were 0.538, 0.519, 0.626, and 0.599, respectively. Further examination of subgroups showed that the correlation between WWI and AS remained consistent and positive. The results of this study show that there is a significant positive correlation between WWI and BaPWV in patients with T2DM. Additionally, compared to other obesity indices, WWI is associated with a higher risk of AS. It may be a potentially simple and effective tool for assessing AS in clinical practice.

**Supplementary Information:**

The online version contains supplementary material available at 10.1038/s41598-025-17715-6.

## Introduction

Type 2 diabetes mellitus (T2DM) is a complex chronic metabolic disease characterized by insulin resistance and insufficient insulin secretion. T2DM not only predisposes individuals to cardiovascular diseases (CVD), but also leads to various complications including neurological disorders^1,2^. Among these, CVD deserves special attention as it is one of the leading causes of death in patients with T2DM. Arterial stiffness (AS) begins before the onset of CVD and causes significant pathophysiological damage, thus exacerbating the deterioration of T2DM^3^. Studies have shown that patients with T2DM commonly exhibit hyperinsulinemia, which leads to increased levels of triglycerides and very low-density lipoproteins in the circulation, thereby accelerating the development of AS^4,5^. Additionally, patients with T2DM often experience hepatic lipid metabolic disorders, which not only promote lipid synthesis but also inhibit β-oxidation of fatty acids. This metabolic imbalance intensifies the state of oxidative stress in the body, further promoting the progression of AS^6–8^.Brachial-ankle pulse wave velocity (BaPWV) measurement is commonly used to assess evaluate AS. It is accurate, simple, non-invasive, and demonstrates high consistency. At present, most BaPWV measurements have been widely applied in the research and clinical evaluation of AS^9,10^. Exploring the risk factors associated with AS and interventions can effectively reduce cardiovascular complications in individuals with diabetes. This brings many benefits to human health and reduces the burden of related diseases on both individuals and society.

T2DM is usually accompanied by overweight or obesity^11^. The prevalence of AS increases significantly when these two diseases coexist^12^. Anthropometry is the most common and inexpensive method for assessing body composition^13^. Therefore, anthropometric indicators related to obesity have received increased attention.

Body mass index (BMI) and waist circumference (WC) are the most commonly used traditional obesity^14^. However, BMI does not differentiate between muscle and fat mass, nor does it reflect fat distribution^15^. WC is an important measure of abdominal fat accumulation and is closely related to BMI^14^. To compensate for the limitations of traditional obesity indices, many new indices based on simple anthropometric dimensions have been developed in the last few years. New anthropometric indices, including a body size index (ABSI), body roundness index (BRI), and weight-adjusted waist index (WWI), are more effective than traditional indices in identifying obesity-related metabolic and chronological diseases^16–19^. Recent research has indicated a strong connection between ABSI, BRI and AS^20^. The WWI was newly developed as an index of obesity. It emphasizes the strengths of WC, diminishes those associated with BMI, and primarily reflects central obesity unrelated to weight^19^. It has been validated for predicting CVD and T2DM^18,21^. There is limited research on the connection between WWI and AS risk, and it is not yet established whether there is a definite association.

This research sought to explore the connection between WWI and increased AS in individuals with T2DM and to evaluate whether WWI predictive capabilities surpass those of several other indices in identifying increased AS.

## Materials and methods

### Sources and selection of research subjects

The subjects of this study all originated from diabetic patients treated at the Changde First People’s Hospital branch of the National Metabolic Disease Standardization Management Cente, during the period from May 2020 to January 2022. Diabetes was defined as having a fasting plasma glucose level of ≥ 7.0 mmol/L, a 2-h plasma glucose level of ≥ 11.1 mmol/, an HbA1c level of ≥ 6.5%, or a self-reported previous diagnosis by health care professionals. The subjects of this study were solely recruited from individuals aged 20–70 years T2DM (*n* = 1647). Exclusion criteria included individuals aged below 20 or above 75 years (*n* = 18), those with gestational diabetes mellitus (*n* = 15), a history of cardiovascular disease (including coronary heart disease, myocardial infarction, heart failure, atrial fibrillation, and stroke) (*n* = 63), stroke, malignant tumors (*n* = 10), and those with missing data on BaPWV, height, and weight (*n* = 86). Additionally, 49 participants were excluded due to an ankle-brachial index (ABI) < 0.9 or > 1.3, as severe atherosclerotic stenosis in the lower-extremity arteries could affect the recording and precision of BaPWV^22^. Finally, 1406 patients were enrolled in this study.

### Anthropometric, clinical and social demographic parameters

Information was collected from subjects through a digital medical record system and surveys, which covered details such as sex, age, smoking, drinking, antihypertensive, hypoglycemic, hypolipidemic medication use, and physical activity. Anthropometric measurements were performed by a specialized endocrinology nurse. Patients were measured for height and weight while wearing light clothing and standing upright without shoes. WC was measured with a tape measure at the midpoint between the inferior border of the costal arch and iliac crest in the mid-axillary line. Blood pressure was measured using an automated electronic sphygmomanometer in a seated position and averaged over three measurements. Smoking status were divided into current daily smokers, former smokers, and never smokers. Drinking was classified as daily, occasional, or never drinking. Physical activity was characterized as moderate to vigorous physical activity, exceeding 150 min/week^23^. Abdominal obesity was defined as WC ≥ 90 cm in males and WC ≥ 85 cm in females^24^.

Each subject fasted for 10–12 h before biochemical index tests were performed, and venous blood specimens were collected from the forearms. Fasting glucose (FPG), 2-hour postprandial plasma glucose (2hPG), total cholesterol (TC), triglyceride (TG), high-density lipoprotein cholesterol (HDL-C), low-density lipoprotein cholesterol (LDL-C), aspartate aminotransferase (AST), alanine aminotransferase (ALT), and creatinine (CR) were measured using automated hematology analyzers (model XE-2100; Sysmex Co., Kobe, Japan) using a standard enzymatic method. Glycosylated hemoglobin (HbA1c) was measured using high-performance liquid chromatography (TOSOH HLC723G8 automatic HbA1C analyzer). The TyG index was determined by taking the natural logarithm of the product of TG (mg/dL) and FPG (mg/dL), divided by 2. eGFR was derived from the CKD-EPI equation^25^.

- BMI^14^, ABSI^16^, BRI^17^ and WWI^18^ formula calculation.

$$\:BMI=\frac{weight}{{height}^{2}}$$

$$\:ABSI=\frac{WC}{({BMI}^{2/3}\times\:{height}^{1/2})}$$

$$\:BRI=364.2-365.5\times\:\sqrt{1-\frac{{\left(\frac{WC}{2\pi\:}\right)}^{2}}{(0.5\:{height)}^{2}}}\:$$

$$\:WWI=\frac{WC}{\sqrt{weight}}$$

- Measurement of BaPWV and definition of AS.

All participants underwent measurements according to the same standards, with all measurements conducted by the same individual. The procedure was performed by a specially trained nurse utilizing the mronColin HBP-8000 device (Omron Healthcare, China). Prior to measurements, participants were instructed to rest in a seated position for 5 to 10 min in a room maintained at a temperature between 22 °C and 25 °C. They were required to wear light clothing and lie supine on the examination table, remaining quiet throughout the measurement process. Cuffs were applied to both the arms and ankles, with the lower edge of the arm cuff positioned 2 to 3 cm above the antecubital crease and the lower edge of the ankle cuff placed 1 to 2 cm above the medial malleolus. The measurement method for BaPWV involved calculating the distance from the sternum to the ankle based on the participant’s height, divided by the time taken for the pulse wave to travel between the forearm and the ankle^26^. ABI was defined as the ankle-to-forearm systolic blood pressure (SBP) ratio. BaPWV measurements were the same in all subjects, this study used the average of the bilateral BaPWV values for ease of analysis. In this study, AS was defined as an elevated BaPWV ≥ 1400 cm/s^27^.

### Statistical analysis

Continuous variables are represented as averages with standard deviations (SD) or medians with ranges; classified variables are represented as the number of participants (%). Group differences were assessed using analysis of variance for normally distributed data, Kruskal-Wallis H test for skewed data, and chi-square or Fisher’s exact test for categorical variables. The ABSI values were too small; therefore, they were expanded by a factor of 100 and labeled as changing every 0.01 units. Second, the Pearson correlation test was performed to test the correlation between the obesity indices (BMI, WC, ABSI, BRI, and WWI) and BaPWV. Third, multivariate linear regression analyses were used to assess the correlations [effect sizes (β) and 95% confidence intervals (CI)] between the five obesity indices and BaPWV. A smoothed curve was utilized to display the relationship between obesity measures and BaPWV. Fourth, using three different sets of adjusted models, multivariate logistic regression was employed to examine the relationship between obesity indices and increased AS by calculating the adjusted odds ratios (ORs) and 95% CIs. Model I was unadjusted; Model II was adjusted for age and sex; and Model III was adjusted for age, sex, smoking, drinking, physical activity, SBP, DBP, medication use (antihypertensive, hypoglycemic, and hypolipidemic medication use), duration of diabetes, FPG, 2hPG, AST, ALT, TC, TG, HDL-C, LDL-C, CR, eGFR, and ABI. Fifth, the effectiveness of every obesity index was assessed through receiver operating characteristic (ROC) analyses. Subgroup analyses of outcomes were conducted using interaction tests. Statistical analyses with R version 4.0.3 and Empower States 4.1. Statistically significant results were determined for *P*-values less than 0.05.

## Results

### Clinical characteristics of subjects

The characteristics of 1406 patients diagnosed with T2DM are displayed in Table 1. Mean age was 52.23 ± 10.66 years. The average BaPWV was (1611.98 ± 318.81) cm/s. The high BaPWV group, in contrast to the normal BaPWV group, consisted of older individuals who were more prone to unhealthy lifestyle choices such as smoking and drinking. The obesity indices (BMI, WC, ABSI, BRI, and WWI) and health indicators (HDL-C, HbA1c, eGFR, and CR) showed significantly elevated levels, with the exception of FPG, TG, TC, and LDL-C (*P* > 0.05). No notable variances were observed in the utilization of medications (specifically those for lowering lipids and glucose) or levels of physical activity.

### Pearson correlation analyses between obesity indices and BaPWV

In Table 2, the relationship between obesity markers in individuals with T2DM and their association with BaPWV is displayed. There was strong correlations were observed between WC and BMI, BRI and WC, BRI and BMI, BRI and WWI, and ABSI and WWI (correlation coefficients ranged from 0.775 to 0.871, *P* < 0.001). Moderate strength correlation between ABSI and WC, ABSI and BRI, WWI, and WC (correlation coefficients range 0.447–0.536, *P* < 0.001). However, there were weaker correlations between WWI and BMI, ABSI, and BMI (correlation coefficients ranged from 0.201 to 0.310, *P* < 0.001). In general, there was a notable connection among all the anthropometric measures of obesity. In addition, BaPWV, as a representative AS, was significantly correlated with all anthropometric obesity indices. Among these indices, WWI exhibited the strongest correlation with BaPWV (*r* = 0.332, *P* < 0.001), followed by ABSI (*r* = 0.269, *P* < 0.001), whereas BMI showed the weakest correlation (*r* = 0.031, *P* < 0.001).

### Linear relationship between obesity indices and BaPWV

**Fig. 1:**
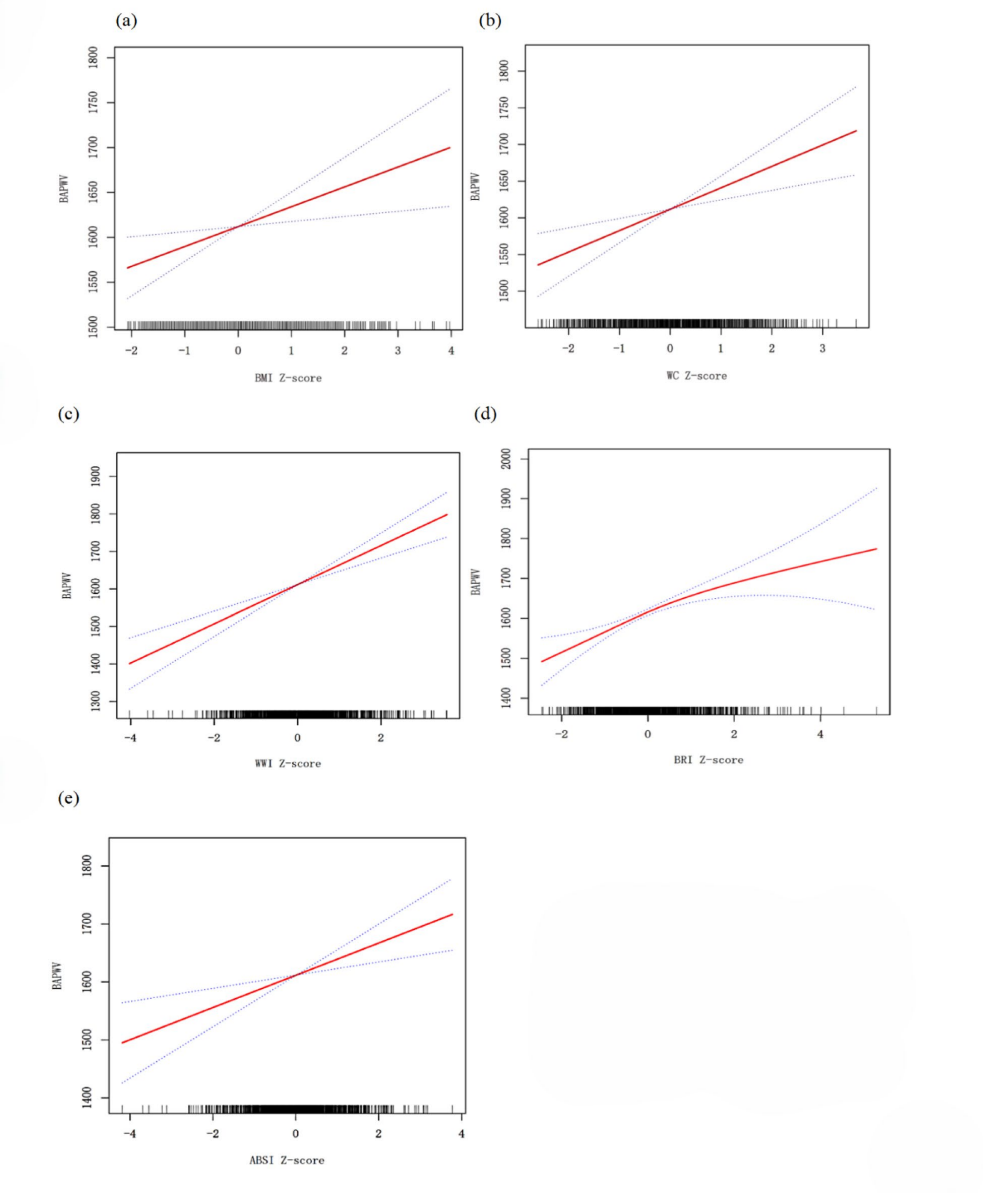
Dose–response relationship between five obesity indices and growth of BaPWV. (a) BMI, (b) WC, (c) WWI, (d) BRI, (e) ABSI. Each plot was adjusted for age, sex, smoking, drinking, physical activity, SBP, DBP, antihypertensive, hypoglycemic, hypolipidemic medications use, duration of diabetics, HbA1c, FPG, 2hPG, TG, HDL-C, TC, LDL-C, ABI, ALT, AST, CR, eGFR.

In the regression analysis, we used the Z-scores of obesity indices. After adjusting for potential confounding factors, multivariate linear regression analysis revealed a positive correlation between BaPWV and all measures of obesity (Table 3). For each SD increase in WWI, BaPWV increased by 48.59 cm/s (95% CI: 31.66–65.52). The β per SD increase of BRI, ABSI, WC, and BMI were 37.83 (22.10-53.56), 27.22 (10.81–43.63), 24.59 (7.81–41.37), 23.05 (6.64–39.47). The positive linear relationship between BMI, WC, ABSI, BRI, WWI, and BaPWV was further confirmed through smooth curve fitting. (Fig. 1)

### Associations between obesity indices with increased prevalence of increased AS

Multivariable logistic regression fully adjusted for selected variables showed (Model III) that the risk of increased AS increased by 44% per SD increase in WWI (OR = 1.44, 95% CI: 1.21–1.70) and the risk of increased AS increased by 35% per SD increase in BRI (OR = 1.35, 95% CI: 1.15–1.58). The ORs per SD increase of ABSI, WC, and BMI were 1.20 (1.02–1.40), 1.19 (1.01–1.39), and 1.19 (1.02–1.39), respectively. Compared with other obesity indices, WWI was more strongly correlated with the prevalence of increased AS. (Table 4)

### ROC analysis of five obesity indices for identifying increased AS

**Fig. 2:**
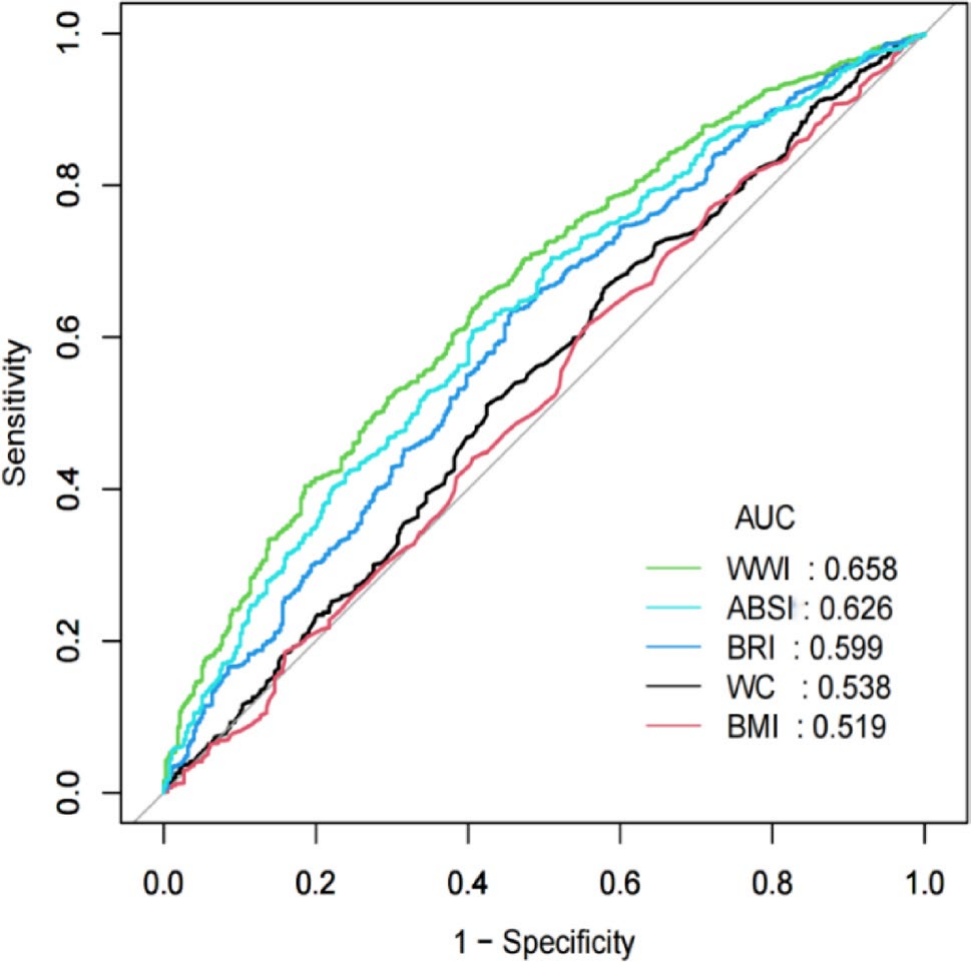
Receiver operating characteristic analysis of five obesity indices for predicting AS. AS, arterial stiffness; BMI, body mass index; WC, waist circumference; WWI, weight-adjusted waist index; ABSII, a body shape index; BRI, body round index; ROC, Receiver-operating-characteristic; AUC, Area under the curve.

The results showed that all five obesity indices were useful indicators for identifying increased AS risk in T2DM patients (Table 5; Fig. 2). Compared with WC (AUC = 0.538), BMI (AUC = 0.519), BRI (AUC = 0.599), ABSI (AUC = 0.626), WWI performed the optimal performance to predict increased AS risk in T2DM subjects (AUC = 0.659, 95% CI:0.627–0.690). The increased AS risk with an elevated obesity index was also compared. It was found that increased AS risk in T2DM subjects increased with an increasing obesity index. The cut-off value we obtained was an important turning point, especially as WWI exceeded this threshold value, and increased AS risk in T2DM subjects almost doubled (Supplementary Table 1). In order to comprehensively evaluate the diagnostic utility of WWI, we also conducted a comparison of AUC (Supplementary Table 4).

### Sensitivity analysis

Increased AS was redefined according to BaPWV ≥ 1800 cm/s. Multivariate logistic analysis (Supplementary Table 2) and ROC curve analysis (Supplementary Table 3) were performed again. The study results suggest that WWI is the best anthropometric obesity index for assessing the risk of increased AS in T2DM patients. The continuous variable of WWI was categorized into quartiles. After fully adjusting for confounders (Model III), WWI was significantly and positively associated with AS. With increasing WWI, AS risk increased by 32% in Q2, 41% in Q3, and 126% in Q4 compared to Q1 (WWI < 10.76) (*P* for trend < 0.001). In contrast to low levels of WWI (< 11.58), high levels of WWI (≥ 11.58) were associated with a notably greater risk of increased AS (OR: 1.84, 95% CI: 1.26–2.69, *P* = 0.002). (Table 6)

### Subgroup analysis

Subgroup analysis was conducted to assess whether the relationship between WWI and AS is stable across different population settings. Stratification was performed based on sex, age (< 50 and ≥ 50 years), SBP (< 140 and ≥ 140 mmHg), DBP (< 90 and ≥ 90 mmHg), BMI (< 24, 24–28, ≥ 28 kg/m2), HBA1c (< 7 and ≥ 7%), duration of diabetes (< 5 and ≥ 5 years), smoking (yes/no), alcohol (yes/no), and physical activity (yes/no). The results indicated that the association between WWI and AS did not exhibit dependency. As shown in Table 7, none of the stratifications including gender, age, BMI, SBP, and DBP status significantly affected the positive correlation between WWI and AS (all interaction *P* > 0.05). The positive correlations across different subgroups were robust. For example, for every 1 SD increase in WWI, the risk of AS may increase by 22% among those with good glycemic control (HBA1c < 7%), and this relationship remains significant in those with HBA1c ≥ 7% (OR = 1.61, 95% CI: 1.30-2.00).

## Discussion

This cross-sectional study explored the association between WWI and increased AS, in addition to comparing WWI, BMI, WC, ABSI, and BRI to provide a more accurate diagnosis. This study observed that five obesity indices were significantly associated with BaPWV. Compared with other obesity indices, WWI was a better obesity index for predicting increased AS risk in T2DM patients.

CVD is not only the major cause of death in T2DM^28^, but also a major component of healthcare expenditure for diabetics (20%−49%)^29^. Therefore, identifying and preventing CVD at an early stage can reduce the burden on individuals with T2DM and the society.

AS is a physiological alteration observed in the initial stages of CVD that includes decreased elasticity of the arteries and abnormalities in the structure of the arteries, with changes in arterial elasticity occurring earlier than structural abnormalities^26,30^. In the initial stages of AS, it is easy to overlook when there are no accompanying symptoms. Therefore, early detection of changes in arterial elasticity is crucial to enable timely clinical intervention and prognosis of the arterial structure.

In this study, BaPWV ≥ 1400 cm/s was prevalent in T2DM patients, with a prevalence rate of 73.19%. The results closely with the conclusions of Kun et al.‘s research, despite variations in sample sizes^31^. Obesity is an individual AS risk factor, particularly with excessive visceral obesity^32,33^. Therefore, identifying a feasible obesity diagnostic tool for AS is crucial.

BMI and WC are the most widely available body measurements for assessing obesity. BMI is not a precise indicator of body fat and does not account for the distribution of muscle and fat, nor does it account for variations in body fat based on gender and race^15,34^. WC is a more accurate reflection of the distribution of body fat and a better predictor of health risks associated with obesity than BMI^35^. WC is strongly correlated with BMI, which indicates that high levels of WC may also be caused by high muscle mass rather than by high fat mass^36^. Despite multiple clinical studies showing a strong connection between BMI and WC and CVD, there is still debate about accurately predicting the risk of obesity-related CVD and mortality^37^. One reason for this is that these anthropometric measurements often fail to distinguish between muscle and fat mass, making it difficult to accurately evaluate metabolic health based on a single index due to varying body compositions. In recent years, there has been a constant development of novel anthropometric measurements. The ABSI and BRI are the most widely used anthropometric indices that have been proposed as alternatives to BMI and WC^38,39^. The ABSI is a novel obesity index based on the allometric principle, which standardizes WC using BMI and height^38^. ABSI is positively related to visceral fat and is a superior predictor of premature mortality compared to BMI and WC^16^. ABSI was identified as a crucial obesity measurement for evaluating AS in individuals with T2DM in the research^40^. ABSI attenuated the effects of BMI and height on WC, but ABSI means were highly clustered and had small variance, complex formulas, and racial heterogeneity. The BRI is a different measure of obesity that can better estimate the amount of body fat and visceral fat compared to BMI and WC^39^. Prior research has explored the correlation between measures of obesity, including BMI, WC, ABSI, and BRI, and AS, as well as their effectiveness in predicting AS risk^32,41,42^, and BRI was shown to have the best ability to identify AS in a cross-sectional study in China. The results obtained (BRI = 0.599) were slightly lower than those reported by Zhang et al. (BRI = 0.631), which may be due to inconsistencies in the study population and sample sizes^41^. However, BRI is complicated to calculate and easily affected by subjectivity, is not easy to popularize, has limited practical application value, and is not convenient for routine examination in clinics and daily life.

Given the limitations of traditional and novel anthropometric indices, there is a need for a better, more convenient, and economically effective comprehensive obesity index to predict cardiovascular-metabolic disease. Park developed the WWI, a new anthropometric index of obesity that emphasizes the strengths of WC, diminishes those associated with BMI, and primarily reflects central obesity unrelated to weight^19^. Unlike BMI, which focuses solely on the correlation between weight and height, WWI emphasizes that WC more accurately captures the risk associated with central obesity. Compared to the ABSI and BRI, WWI calculations are simpler and more readily available. Furthermore, WWI can distinguish between fat and muscle mass, which is positively related to visceral fat and inversely related to abdominal muscle^43,44^. A recent multiracial atherosclerosis study suggested that racial differences in the distribution of WWI are small and suitable for assessing body fat content and muscle mass in multiracial populations^45^.

As an emerging index, WWI performs excellently in predicting CVD risk and has a strong association with various risk factors for CVD. Recent research has discovered a positive correlation between WWI levels and the presence of vascular calcification^46^ and hypertension^47^. Several prospective studies have shown that higher WWI levels significantly increased the risk of CVD death^21,48^. In addition, WWI is an independent predictor of heart failure^49^, left ventricular hypertrophy^50^, and stroke^51^. These studies suggest that WWI could be a significant predictor of increased AS risk.

In multivariate logistic analyses, we performed Z-score transformations on five obesity indices and observed their effects on the BaPWV effect size, ORs, and 95% CI. The study results were consistent with those of the ROC analysis. WWI was consistently positively associated with BaPWV both before and after model adjustment (β = 48.59, 95% CI: 31.66–65.52). This is similar to the positive association between WWI and BaPWV found by Xiong et al. in a Chinese hypertensive patient (β = 57.98)^52^. Although other obesity indices also performed well in predicting an increased AS risk, WWI was the best predictor (AUC = 0.689). The findings from this research align with the outcomes of earlier studies, with an AUC range of 0.651–0.677^46,53^. WWI ≥ 11.40 greatly increases the risk of increased AS in T2DM subjects, which is consistent with early studies on the adverse effects of WWI on cardiovascular health (WWI: 11.20-11.47)^21,53^.

Multivariate logistic regression adjusted for known confounders such as age, sex, SBP, DBP, duration of diabetes, medication use, ALT, AST, smoking, and alcohol consumption^54,55^. The results showed that WWI was independently positively associated with AS. AS is closely linked to nonalcoholic fatty liver disease (NAFLD) according to studies^56,57^. ALT and AST ≥ 40 IU/L used as screening thresholds for NAFLD^58,59^. Subgroup analysis categorized ALT and AST levels as ≥ 40IU/L and < 40IU/L. WWI was positively correlated with AS risk in both subgroups, with no interaction detected between subgroups based on the interaction test. Studies have shown a strong link between the TyG index and liver fat content, as well as its ability to predict NAFLD with high accuracy^59^. Similarly, no interaction was found between TyG subgroups. Therefore, close monitoring of patients with WWI is crucial to mitigate AS risk, regardless of liver involvement.

There are several possible explanations for the higher WWI and increased AS. First, WWI is positively related to visceral fat and inversely related to muscle mass^43,44^. High levels of WWI signal excessive fat storage and loss of muscle mass. The presence of too much fat and infiltration of macrophages in fat tissue can disrupt the secretion of adipocytokines, resulting in lower levels of adiponectin and higher levels of interleukin 6^60^. The disruption of adipokines results in endothelial dysfunction, vascular remodeling, and plays a crucial role in the initial phases of AS^61,62^. Furthermore, besides altered adipocytokine secretion, fat deposition and infiltration also contribute to insulin resistance by disrupting insulin signaling and glucose homeostasis^63^. In insulin-resistant states, elevated plasma asymmetric dimethylarginine (ADMA) levels reduce plasma nitric oxide (NO) synthesis, which is essential for maintaining vascular tone and structure. In addition, it induces endothelial nitric oxide synthase (eNOS) uncoupling and enhances reactive oxygen species (ROS) production, which can accelerate chronic inflammation and oxidative stress, leading to increased AS^64–67^. Second, WWI is closely associated with many diseases (T2DM^18^, hypertension^44^, metabolic syndrome^68^, which accelerates the development of AS. Third, WWI has been shown to increase with age and may reflect changes in body composition changes^43^. This may partially explain the association between WWI and AS. As age increases, body fat is redistributed and more likely to accumulate in the internal organs as well as in the abdomen^69^. Increased visceral adiposity directly promotes systemic microinflammation, which is a key pathway in AS progression^43,70^.

The results have clinical and public health importance. It demonstrates that the WWI is a predictor of increased AS risk in patients with T2DM. WWI is simple to calculate, easy to obtain, and can be widely used in hospitals at all levels. WWI can be used as a screening tool, with WWI ≥ 11.40 alerting clinicians to those at risk for increased AS. Additionally, WWI is economically inexpensive, highly reproducible, and provides a practical method for screening for increased AS risk that can reduce healthcare expenditure, particularly in areas where healthcare resources are relatively scarce.

### Study strengths and limitations

This study’s main advantage lies in comparing various anthropometric measurements and their ability to predict increased AS in individuals with T2DM. Limitations of this study should also be taken into account. First, although this study provides insights into patients with T2DM, the generalizability of the findings to other populations, such as those with prediabetes or non-diabetic individuals, may be limited due to potential differences in pathophysiological and metabolic characteristics. Second, although the regression analysis was adjusted for numerous key parameters, it is possible that the outcomes were affected by additional confounding variables not included in the adjustment. Finally, the cross-sectional design of this study precluded the establishment of causality.

## Conclusion

The results of this study show that there is a significant positive correlation between WWI and BaPWV in patients with T2DM. Additionally, compared to other obesity indices, WWI is associated with a higher risk of AS. It may be a potentially simple and effective tool for assessing AS in clinical practice. However, further studies are still needed to validate our findings.

**Table 1:** Baseline characteristics of the participants.

Parameter	Total	No-AS group (BaPWV < 1400 cm/s)	AS group (BaPWV ≥ 1400 cm/s)	*P*-value
Age(years)	52.23 (10.66)	45.97 (9.92)	54.55 (9.98)	< 0.001
DBP (mmHg)	83.37 (11.14)	80.15 (10.21)	84.54 (11.23)	< 0.001
SBP (mmHg)	135.56 (19.21)	124.12 (14.92)	139.74 (18.88)	< 0.001
Course of diabetes(month)	38.00 (5.00–96.00)	24.00 (1.00–61.00)	48.00(8.50–108.00)	< 0.001
HbA1c (%)	8.33 (2.21)	8.59 (2.44)	8.23 (2.11)	0.007
FPG (mmol/L)	8.67 (3.15)	8.85 (3.37)	8.61 (3.06)	0.193
2hPG (mmol/L)	13.16 (4.57)	12.88 (4.73)	13.27 (4.51)	0.157
ALT (U/L)	25.00(17.00-38.75)	26.00(17.25-40.00)	24.00(17.00–38.00)	0.358
AST (U/L)	26.16 (13.34)	25.83 (13.31)	26.26 (13.34)	0.591
CR (umol/L)	64.00 (22.17)	60.57 (15.53)	65.28 (24.06)	< 0.001
eGFR(mL/min/1.73m2)	106.53 (20.29)	114.95 (16.83)	103.41 (20.60)	< 0.001
TG (mmol/L)	1.82 (1.29–2.81)	1.75 (1.25–2.72)	1.88 (1.31–2.84)	0.126
TC (mmol/L)	4.95 (1.25)	4.92 (1.32)	4.96 (1.22)	0.656
HDL-C (mmol/L)	1.26 (0.33)	1.23 (0.32)	1.27 (0.33)	0.026
LDL-C (mmol/L)	2.79 (0.90)	2.77 (0.87)	2.79 (0.91)	0.652
BaPWV (cm/s)	1611.98 (318.81)	1286.30 (86.03)	1731.23 (287.82)	< 0.001
ABI	1.12 (0.08)	1.09 (0.07)	1.13 (0.07)	< 0.001
WC (cm)	67.0 (12.4)	68.7 (12.9)	66.3 (12.2)	0.001
BMI (kg/m2)	25.57 (3.41)	25.46 (3.55)	25.61 (3.35)	0.450
WWI	11.18 (0.66)	10.91 (0.63)	11.27 (0.68)	< 0.001
BRI	4.72 (1.24)	4.40 (1.19)	4.82 (1.25)	< 0.001
ABSI×100	8.28 (0.40)	8.14 (0.41)	8.32 (0.42)	< 0.001
TyG	2.06 (0.74)	2.02 (0.79)	2.07 (0.72)	0.297
Sex (n, %)				0.040
Male	818 (58.18%)	236 (62.60%)	582 (56.56%)	
Female	588 (41.82%)	141 (37.40%)	447 (43.44%)	
Drinking status (n, %)				0.030
Never	956 (67.99%)	243 (64.46%)	713 (69.29%)	
Occasional	104 (7.40%)	39 (10.34%)	65 (6.32%)	
Everyday	346 (24.61%)	95 (25.20%)	251 (24.39%)	
Smoking status (n, %)				0.026
Never	884 (62.87%)	220 (58.36%)	664 (64.53%)	
Former	113 (8.04%)	27 (7.16%)	86 (8.36%)	
Current everyday	409 (29.09%)	130 (34.48%)	279 (27.11%)	
Physical activity (n, %)				0.242
No	302 (21.48%)	89 (23.61%)	213 (20.70%)	
Yes	1104 (78.52%)	288(76.39%)	816 (79.30%)	
Hypoglycemic medications (n, %)				0.172
No	212 (15.08%)	65 (17.24%)	147 (14.29%)	
Yes	1194 (84.92%)	312 (82.76%)	882 (85.71%)	
Hypolipidemic medications (n, %)				0.318
No	1227 (87.27%)	335 (88.86%)	892 (86.69%)	
Yes	179 (12.73%)	42 (11.14%)	137 (13.31%)	
Antihypertensive medications (n, %)				< 0.001
No	1027 (73.04%)	333 (88.33%)	694 (67.44%)	
Yes	379 (26.96%)	44 (11.67%)	335 (32.56%)	

SBP, systolic blood pressure; DBP, diastolic blood pressure; HbA1c, glycated hemoglobin; FPG, fasting plasma glucose; 2hPG, 2-hour postprandial plasma glucose; ALT, alanine aminotransferase; AST, aspartate aminotransferase; TG, triglyceride; TC, total cholesterol; HDL-C, high-density lipoprotein cholesterol; LDL-C, low-density lipoprotein cholesterol; CR, creatinine; eGFR, estimated glomerular filtration rate; BMI, body mass index; WC, waist circumference; WWI, weight-adjusted waist index; ABSI, a body shape index; BRI, body roundness index; BaPWV, brachial-ankle pulse wave velocity; ABI, ankle-brachial index; AS, arterial stiffness.

**Table 2:** Pearson correlation coefficients between obesity indices and bapwv.

Variables	BaPWV	WC	BMI	WWI	BRI	ABSI
WC	0.094*	1				
BMI	0.031*	0.871*	1			
WWI	0.332*	0.539*	0.310*	1		
BRI	0.207*	0.874*	0.834*	0.775*	1	
ABSI	0.269*	0.447*	0.201*	0.857*	0.495*	1

*P < 0.001.

WC, waist circumference; BMI, body mass index; WWI, weight-adjusted waist index; ABSI, a body shape index; BRI, body roundness index; BaPWV, Brachial-ankle pulse wave velocity.

**Table 3:** Multiple linear regression analysis of relationship between obesity indices and bapwv.

	Model I β (95% CI) *P*-value	Model II β (95% CI) *P*-value	Model III β (95% CI) *P*-value
WC Z- score	30.44 (13.62, 47.26) < 0.001	45.78 (30.28, 61.28) < 0.001	24.59 (7.81, 41.37) 0.004
BMI Z-score	10.02 (−6.64, 26.69) 0.239	43.97 (28.87, 59.06) < 0.001	23.05 (6.64, 39.47) 0.006
WWI Z-score	110.47 (94.02, 126.91) < 0.001	63.52 (46.62, 80.42) < 0.001	48.59 (31.66, 65.52) < 0.001
BRI Z- score	66.69 (50.23, 83.16) < 0.001	54.61 (39.89, 69.33) < 0.001	37.83 (22.10, 53.56) 0.001
ABSI Z-score	91.31 (74.20, 108.42) < 0.001	35.94 (18.96, 52.93) < 0.001	27.22 (10.81, 43.63) < 0.001

Model I was non adjusted. Model II was adjusted for age and sex. Model III was adjusted for age, sex, smoking, drinking, physical activity, SBP, DBP, antihypertensive, hypoglycemic, and hypolipidemic medication use, duration of diabetes, HbA1c, FPG, 2hPG, ALT, AST, TG, HDL-C, TC, LDL-C, ABI, CR, and eGFR.

BaPWV, Brachial-ankle pulse wave velocity; BMI, body mass index; WC, waist circumference; WWI, weight-adjusted waist index; ABSI, a body shape index; BRI, body round index; β, effect size; CI, confidence interval.

**Table 4:** Logistic regression analysis of association between obesity index and increased prevalence of increased AS.

	Model I OR (95% CI) *P*-value	Model II OR (95% CI) *P*-value	Model III OR (95% CI) *P*-value
WC Z-score	1.15 (1.02, 1.30) 0.025	1.29 (1.13, 1.48) < 0.001	1.19 (1.01, 1.39) 0.035
BMI Z-score	1.05 (0.93, 1.18) 0.457	1.31 (1.15, 1.50) < 0.001	1.19 (1.02, 1.39) 0.025
WWI Z-score	1.89 (1.64, 2.17) < 0.001	1.50 (1.29, 1.76) < 0.001	1.44 (1.21, 1.70) < 0.001
BRI Z-score	1.45 (1.27, 1.65) < 0.001	1.43 (1.25, 1.65) < 0.001	1.35 (1.15, 1.58) < 0.001
ABSI Z-score	1.62 (1.41, 1.85) < 0.001	1.22 (1.05, 1.41) 0.010	1.20 (1.02, 1.40) 0.026

Model I was non adjusted. Model II was adjusted for age and sex. Model III was adjusted for age, sex, smoking, drinking, physical activity, SBP, DBP, antihypertensive, hypoglycemic, and hypolipidemic medication use, duration of diabetes, HbA1c, FPG, 2hPG, ALT, AST, TG, HDL-C, TC, LDL-C, ABI, CR, and eGFR.

AS, arterial stiffness; BMI, body mass index; WC, waist circumference; WWI, weight-adjusted waist index; ABSI, a body shape index; BRI, body round index; OR, odds ratio; CI, confidence interval.

**Table 5:** ROC analysis of the five obesity indices for predicting increased AS.

	AUC	95%CI low	95%CI upp	cut-off	Specificity	Sensitivity	PPV	NPV
WC	0.538	0.504	0.573	91.05	0.576	0.510	0.766	0.301
BMI	0.519	0.484	0.553	24.35	0.440	0.618	0.751	0.297
WWI	0.659	0.627	0.690	11.02	0.584	0.651	0.810	0.380
BRI	0.599	0.566	0.633	4.34	0.544	0.634	0.791	0.352
ABSI*100	0.626	0.594	0.659	8.22	0.594	0.608	0.804	0.357

AS, arterial stiffness; BMI, body mass index; WC, waist circumference; WWI, weight-adjusted waist index; ABSI, a body shape index; BRI, body round index; ROC, receiver operating characteristic; AUC, area under the curve; CI, Confidence interval; NPV, negative predictive value; PPV, positive predictive value.

**Table 6:** Association between WWI and increased AS in different models.

	Model I	Model II	Model III
WWI Z-score	1.89 (1.64, 2.17) < 0.001	1.50 (1.29, 1.76) < 0.001	1.44 (1.21, 1.70) < 0.001
WWI quartile			
Q1(< 10.76)	1	1	1
Q2(10.76–11.14)	1.70 (1.24, 2.33) 0.001	1.41 (1.01, 1.97) 0.043	1.32 (0.93, 1.87) 0.121
Q3(11.14–11.58)	2.21 (1.60, 3.07) < 0.001	1.56 (1.10, 2.21) 0.013	1.41 (0.97, 2.04) 0.072
Q4(≥ 11.58)	4.41 (3.04, 6.40) < 0.001	2.46 (1.63, 3.72) < 0.001	2.26 (1.45, 3.52) < 0.001
P for trend	< 0.001	< 0.001	< 0.001
WWI categories			
Q1–3 (< 11.58)	1	1	1
Q4(≥ 11.58)	2.89 (2.07, 4.02) < 0.001	1.87 (1.30, 2.69) < 0.001	1.84 (1.26, 2.69) 0.002

Model I was non adjusted. Model II was adjusted for age and sex. Model III was adjusted for age, sex, smoking, drinking, physical activity, SBP, DBP, antihypertensive, hypoglycemic, and hypolipidemic medication use, duration of diabetes, HbA1c, FPG, 2hPG, ALT, AST, TG, HDL-C, TC, LDL-C, ABI, CR, and eGFR.

AS, arterial stiffness; BMI, body mass index; WC, waist circumference; WWI, weight-adjusted waist index; ABSI, a body shape index; BRI, body round index; OR, odds ratio; CI, confidence interval.

**Table 7:** Subgroup analysis of the association between WWI Z-scores and increased AS.

Subgroup	OR (95% CI)	*P* for interaction
Sex		0.251
Male	1.31 (1.04, 1.65)	
Female	1.57 (1.25, 1.99)	
Age (years)		0.567
< 50	1.47 (1.14, 1.89)	
≥ 50	1.61 (1.31, 1.98)	
DBP (mmHg)		0.637
< 90	2.07 (1.52, 2.83)	
≥ 90	1.88 (1.20, 2.95)	
SBP (mmHg)		0.582
< 140	1.98 (1.45, 2.71)	
≥ 140	2.32 (1.36, 3.95)	
Abdominal obesity		0.359
Yes	1.59 (1.24, 2.04)	
No	1.34 (1.01, 1.79)	
BMI (kg/m2)		0.671
< 24	1.46 (1.12, 1.91)	
≥ 24, < 28	1.35 (1.03, 1.64)	
≥ 28	1.64 (1.13, 2.39)	
TyG		0.706
< 2.03	1.38 (1.11, 1.72)	
≥ 2.03	1.47 (1.15, 1.88)	
ALT (U/L)		0.261
< 40	1.37 (1.14, 1.65)	
≥ 40	1.73 (1.19, 2.52)	
AST (U/L)		0.194
< 40	1.41 (1.18, 1.68)	
≥ 40	2.15 (1.14, 4.03)	
Duration of diabetes (years)		0.545
< 5	1.39 (1.14, 1.70)	
≥ 5	1.54 (1.17, 2.02)	
HbA1c (%)		0.080
< 7	1.22 (0.96, 1.56)	
≥ 7	1.61 (1.30, 2.00)	
Smoking status		0.915
Yes	1.44 (1.18, 1.76)	
No	1.42 (1.07, 1.88)	
Drinking status		0.561
Yes	1.55 (1.14, 2.12)	
No	1.40 (1.16, 1.70)	
Physical activity		0.285
Yes	1.19 (0.81, 1.74)	
No	1.49 (1.24, 1.80)	

Adjusted for age, sex, smoking, drinking, physical activity, SBP, DBP, antihypertensive, hypoglycemic, hypolipidemic medication use, duration of diabetes, HbA1c, FPG, 2hPG, ALT, AST, TG, HDL-C, TC, LDL-C, ABI, CR, and eGFR, except for stratified variables.

AS, arterial stiffness; WWI Z-score, weight-adjusted waist index Z-score; BMI, body mass index. Abdominal obesity was defined as WC ≥ 90 cm in males and WC ≥ 85 cm in females.

## Supplementary Information

Below is the link to the electronic supplementary material.

Supplementary Material 1

Supplementary Material 2

Supplementary Material 3

Supplementary Material 4

## Data Availability

The datasets generated and/or analysed during the current study are not publicly available due the database is writing the article but are available from the corresponding author on reasonable request.

## Abbreviations

BMI: Body mass index
ABSI: A body size index
BRI: Body roundness index
WWI: Weight-adjusted waist circumference index
DBP: Diastolic blood pressure
SBP: Systolic blood pressure
FPG: Fasting plasma glucose
HbA1c: Glycated hemoglobin
TC: Total cholesterol
TG: Triglycerides
HDL-C: High-density lipoprotein cholesterol
LDL-C: Low-density lipoprotein cholesterol
CR: Creatinine
eGFR: Glomerular filtration rate
β: Effect size
OR: Odds ratio
CI: Confidence interval
ROC: Receiver operating characteristic
AUC: Area under the curve
T2DM: Type 2 diabetes mellitus
BaPWV: Brachial ankle pulse wave velocity
CVD: Cardiovascular disease
AS: Arterial stiffness
