# Supplementary material for: Weight adjusted waist index is a superior obesity index for predicting arterial stiffness in type 2 diabetes mellitus
Source: Sci Rep. 2025 Aug 29;15:31859. doi: 10.1038/s41598-025-17715-6 (PMC12397426; doi:10.1038/s41598-025-17715-6)
Supplement: Supplementary file 1 — Supplementary Material 1 [file 41598_2025_17715_MOESM1_ESM.docx]

Supplementary Material

**Weight-adjusted-waist index is a superior obesity index for predicting arterial stiffness in type 2 diabetes mellitus**

**Shijun Gong^1†^, Jing Mao^2†^, Quan Zhou^4^, HaiFeng Zhou^3^, Qin Liu^3^, Sun Ting^3^, Shenglian Gan^3*^**

*** Correspondence:** Shenglian Gan: [ganslghy03@126.com](mailto:ganslghy03@126.com)

**Supplementary Table 1** The tendency of the prevalence of AS as the escalation of five obesity indices.

| Variables | Without AS | With AS | The prevalence of AS (%) | P-value |
| --- | --- | --- | --- | --- |
| WC |  |  |  | 0.004 |
| <91.05 | 217 | 504 | 48.98 |  |
| ≥91.05 | 160 | 525 | 51.02 |  |
| BMI | | |  | 0.047 |
| <24.35 | 166 | 393 | 38.19 |  |
| ≥24.35 | 211 | 636 | 61.81 |  |
| WWI | | |  | <0.001 |
| <11.02 | 219 | 359 | 34.89 |  |
| ≥11.02 | 158 | 670 | 65.11 |  |
| BRI | | |  | <0.001 |
| <4.34 | 197 | 370 | 35.96 |  |
| ≥4.34 | 180 | 659 | 64.04 |  |
| ABSI*100 | | |  | <0.001 |
| <8.22 | 220 | 400 | 38.87 |  |
| ≥8.22 | 157 | 629 | 61.13 |  |

Abbreviations: WC, waist circumference; BMI, body mass index; WWI, weight adjusted waist index; ABSI, a body shape index; BRI, body roundness index; AS, arterial stiffness.
