# Supplementary material for: Weight adjusted waist index is a superior obesity index for predicting arterial stiffness in type 2 diabetes mellitus
Source: Sci Rep. 2025 Aug 29;15:31859. doi: 10.1038/s41598-025-17715-6 (PMC12397426; doi:10.1038/s41598-025-17715-6)
Supplement: Supplementary file 2 — Supplementary Material 2 [file 41598_2025_17715_MOESM2_ESM.docx]

Supplementary Material

**Weight-adjusted-waist index is a superior obesity index for predicting arterial stiffness in type 2 diabetes mellitus**

**Shijun Gong^1†^, Jing Mao^2†^, Quan Zhou^4^, HaiFeng Zhou^3^, Qin Liu^3^, Sun Ting^3^, Shenglian Gan^3*^**

*** Correspondence:** Shenglian Gan: [ganslghy03@126.com](mailto:ganslghy03@126.com)

**Supplementary Table 2** Logistic regression analysis of association between obesity index and increased prevalence of AS (BaPWV ≥1800 cm/s).

|  | Model I | Model II | Model III |
| --- | --- | --- | --- |
|  | OR (95% CI) P-value | OR (95% CI) P-value | OR (95% CI) P-value |
| WC Z-score | 1.20 (1.06, 1.35) 0.005 | 1.36 (1.18, 1.58) <0.001 | 1.21 (1.02, 1.44) 0.027 |
| BMI Z-score | 1.06 (0.94, 1.20) 0.324 | 1.37 (1.19, 1.58) <0.001 | 1.23 (1.04, 1.46) 0.014 |
| WWI Z-score | 2.02 (1.76, 2.33) <0.001 | 1.50 (1.28, 1.76) <0.001 | 1.43 (1.20, 1.70) <0.001 |
| BRI Z-score | 1.48 (1.31, 1.68) <0.001 | 1.43 (1.25, 1.64) <0.001 | 1.33 (1.14, 1.56) <0.001 |
| ABSI Z-score | 1.77 (1.54, 2.04) <0.001 | 1.23 (1.05, 1.43) 0.010 | 1.19 (1.01, 1.41) 0.042 |

Model I was non adjusted. Model II was adjusted for age, sex. Model III was adjusted for age, sex, smoking, alcohol, physical activity, SBP, DBP, antihypertensive, hypoglycemic, hypolipidemic medications use, duration of diabetes, HbA1c, FPG, TG, HDL-C, TC, LDL-C, ABI, CR, eGFR.

Abbreviations: AS, arterial stiffness; BMI, body mass index; WC, waist circumference; WWI, weight adjusted waist index; ABSI, a body shape index; BRI, body round index; OR, odds ratios; CI, confidence interval.
