# Supplementary material for: Weight adjusted waist index is a superior obesity index for predicting arterial stiffness in type 2 diabetes mellitus
Source: Sci Rep. 2025 Aug 29;15:31859. doi: 10.1038/s41598-025-17715-6 (PMC12397426; doi:10.1038/s41598-025-17715-6)
Supplement: Supplementary file 3 — Supplementary Material 3 [file 41598_2025_17715_MOESM3_ESM.docx]

Supplementary Material

**Weight-adjusted-waist index is a superior obesity index for predicting arterial stiffness in type 2 diabetes mellitus**

**Shijun Gong^1†^, Jing Mao^2†^, Quan Zhou^4^, HaiFeng Zhou^3^, Qin Liu^3^, Sun Ting^3^, Shenglian Gan^3*^**

*** Correspondence:** Shenglian Gan: [ganslghy03@126.com](mailto:ganslghy03@126.com)

**Supplementary Table 3** ROC analysis of five obesity indices for predicting AS (BaPWV ≥1800 cm/s).

|  | AUC | 95%CI low | 95%CI upp | cut-off | Specificity | Sensitivity | PPV | NPV |
| --- | --- | --- | --- | --- | --- | --- | --- | --- |
| WC | 0.554 | 0.520 | 0.589 | 88.35 | 0.412 | 0.699 | 0.271 | 0.814 |
| BMI | 0.526 | 0.492 | 0.561 | 25.95 | 0.592 | 0.478 | 0.268 | 0.784 |
| WWI | 0.689 | 0.657 | 0.721 | 11.40 | 0.733 | 0.564 | 0.398 | 0.843 |
| BRI | 0.628 | 0.595 | 0.660 | 4.60 | 0.551 | 0.663 | 0.316 | 0.839 |
| ABSI*100 | 0.648 | 0.615 | 0.682 | 8.25 | 0.524 | 0.699 | 0.315 | 0.847 |

Abbreviations: AS, arterial stiffness; BMI, body mass index; WC, waist circumference; WWI, weight adjusted waist index; ABSI, a body shape index; BRI, body round index; ROC, Receiver-operating-characteristic; AUC, Area under the curve; CI, Confidence interval; NPV, negative predictive value; PPV, positive predictive value.
