# Supplementary material for: Weight adjusted waist index is a superior obesity index for predicting arterial stiffness in type 2 diabetes mellitus
Source: Sci Rep. 2025 Aug 29;15:31859. doi: 10.1038/s41598-025-17715-6 (PMC12397426; doi:10.1038/s41598-025-17715-6)
Supplement: Supplementary file 4 — Supplementary Material 4 [file 41598_2025_17715_MOESM4_ESM.docx]

Supplementary Material

**Weight-adjusted-waist index is a superior obesity index for predicting arterial stiffness in type 2 diabetes mellitus**

**Shijun Gong^1†^, Jing Mao^2†^, Quan Zhou^4^, HaiFeng Zhou^3^, Qin Liu^3^, Sun Ting^3^, Shenglian Gan^3*^**

*** Correspondence:** Shenglian Gan: [ganslghy03@126.com](mailto:ganslghy03@126.com)

**Supplementary Table 4 Comparison of AUC values among WWI、WC、BMI、ABSI、BRI**

|  | NRI | 95%CI low | 95%CI upp | *P* value |
| --- | --- | --- | --- | --- |
| WWI vs WC | -0.148 | -0.215 | -0.082 | <0.001 |
| WWI vs BMI | -0.176 | -0.248 | -0.106 | <0.001 |
| WWI vs ABSI | -0.032 | -0.084 | 0.021 | 0.234 |
| WWI vs BRI | -0.057 | -0.111 | -0.003 | 0.038 |

Abbreviations: AS, arterial stiffness; BMI, body mass index; WC, waist circumference; WWI, weight adjusted waist index; ABSI, a body shape index; BRI, body round index; ROC, Receiver-operating-characteristic; AUC, Area under the curve; CI, Confidence interval; NPV, negative predictive value; PPV, positive predictive value.
